# Bioequivalence of two tacrolimus 1-mg formulations under fasting conditions in healthy subjects: A randomized, two-period crossover trial 

**DOI:** 10.5414/CP203534

**Published:** 2019-12-16

**Authors:** Lalitendu Mohanty, Sumit Bhushan, Bernd Rüttger

**Affiliations:** 1 Clinical Research Department, Panacea Biotec Ltd. New Delhi, India, and; 2 Clinical Research Department, Panacea Biotec Germany GmbH, Munich, Germany

**Keywords:** pharmacokinetic, Prograf, tacrolimus, Tacpan, transplant

## Abstract

Objective: The present study compared the pharmacokinetics of two (1 mg) tacrolimus formulations (test (generic from Panacea) and reference (innovator from Astellas)) after a single-dose administration as per the European Medicine Agency (EMA) guidelines to grant marketing authorization. Materials and methods: This study was a randomized, open-label, balanced, two-treatment, two-period, two-sequences, single-dose, truncated-area, crossover design with a washout period of 19 days between the phases. Healthy subjects aged 18 – 45 years (both inclusive) were included. Eligible subjects received a single oral dose of 5 × 1-mg capsule of tacrolimus either test or reference formulation. Blood samples were collected until 72.00 hours postdose, and peak concentration (C_max_) and area under the curve (AUC_0–72_) were evaluated in whole blood using validated LC-MS/MS. Safety was also assessed in each period. Results: Of 56 subjects enrolled, 52 completed both study periods. The arithmetic mean (SD) C_max_ for the reference and test formulations was 40.62 (11.30) and 46.20 (10.73) ng/mL, and AUC_0–72_ was 348.34 (156.41) and 361.04 (158.71) ng×h/mL, respectively. The geometric least square mean ratio (90% confidence interval (CI)) was 115.07% (90% CI: 109.81, 120.59) for C_max_ and 103.78 (90% CI: 97.40, 110.58) for AUC_0–72_, which fell within the acceptance range as per EMA guidelines for narrow therapeutic index drugs (C_max_: 80.00 – 125.00%; AUC: 90.00 – 111.11%). No serious adverse event was observed. Conclusion: The generic tacrolimus was bioequivalent to the reference formulation, was well tolerated, and provides a well-acceptable alternative to the reference drug. Switching treatment to generic tacrolimus medication may reduce the cost and economic burden of treating transplanted patients.

**What is known about this subject**

- Tacrolimus, a narrow therapeutic index drug, is indicated for prophylaxis of organ rejection in patients receiving allogeneic liver, kidney, or heart transplants due to its immunosuppressant property.
- Generic versions of tacrolimus appear to be safe, and switching from brand-name tacrolimus is widely encouraged.

**What this study adds**

- A generic tacrolimus-containing product, developed by Panacea Biotec, was well tolerated and met the requirements of the European Medicine Agency guidelines for narrow therapeutic index drugs (NTID) in healthy subjects.
- This generic version of tacrolimus could provide a well-accepted alternative to the reference drug.
- This study is being published to increase transparency for these types of analyses and improve the confidence clinicians have regarding generic formulations of NTID medications.

## Introduction 

Transplantation is one of the most suitable therapies in several conditions of end-stage organ failure, such as renal, hepatic, or cardiac failure. Over 20,000 kidney transplant and 7,000 liver transplant procedures were performed in European Union countries in 2017, which significantly improved the quality of life of patients with end-stage organ failure [1]. Many patients who undergo transplantation require lifelong immunosuppression essential for long-term graft survival [2]. 

Tacrolimus, a macrolide immunosuppressant, is indicated in patients receiving allogeneic liver, kidney, or heart transplants as the choice for primary immunosuppression for the prophylaxis of organ rejection [3]. It was first approved in the United States (U.S.) to prevent organ rejection in patients receiving allogeneic liver in 1994 followed by kidney in 1997 and heart in 2006 [4]. The usual oral starting dose is 0.20 – 0.30 mg/kg/day for kidney transplant patients and 0.10 – 0.20 mg/kg/day for liver transplant patients, administered as 2 daily doses every 12 hours [3]. The effect of immunosuppression appears to be mediated by binding to intracellular protein FKBP12, which inhibits the phosphatase activity of calcineurin and further prevents dephosphorylation and translocation of the nuclear factor of activated T cells, resulting in inhibition of T-lymphocyte activation [5]. 

Tacrolimus belongs to the category of drugs with narrow therapeutic index for which small changes in dose or exposure could exhibit serious clinical consequences associated with graft rejection [6]. In addition, tacrolimus belongs to the class of “critical drugs” because of high inter- and intrasubject variability and lack of correlation between the administered dose and blood concentrations or clinical events [7]. Because of such variability and potential for several drug interactions, highly individualized dosing serves as an integral part of tacrolimus therapy [8]. Therapeutic drug monitoring (TDM) helps clinicians in maintaining optimum levels of tacrolimus within their respective therapeutic ranges. 

Introduction of generic immunosuppressants illustrates a significant cost reduction over a 5-year (2008 – 2013) period, which consequently increased their uptake from percentages of 10 – 11% in 2009 to 76 – 80% in 2013 in the U.S. [9]. The reduction in the overall financial burden with generic version of tacrolimus ultimately reduced the cost-related medication nonadherence in transplant recipients, which is essential for long-term graft survival [9]. Also, studies have suggested that transplant patients may be safely switched on to the generic tacrolimus provided that their trough concentrations be closely monitored [10, 11]. Currently, there are at least ten different generic equivalents for tacrolimus that are approved by the European Medicines Agency (EMA) and the U.S. Food and Drug Administration. However, the market share of generic immunosuppressive drugs in a few regions of the world is still very low due to a certain reluctance of a physician to accept tacrolimus generics. In addition, tacrolimus is considered to be a narrow therapeutic window drug that is approved using the standard bioequivalence criteria of 80.00 – 125.00% [12, 13]. The EMA recommends strict bioequivalence standards that require a narrower acceptance criterion (90.00 – 111.11%) for the area under the curve (AUC) as opposed to conventional bioequivalence acceptance criteria of 80.00 – 125.00% [14]. 

The rate of absorption of tacrolimus is variable with peak blood and plasma concentrations (C_max_) being reached in 0.5 – 6 hours with different formulations [8]. After oral administration of tacrolimus capsules, time to peak concentrations of tacrolimus in whole blood was observed at ~ 1 – 3 hours and mean half-life (T_1/2_) at ~ 43 hours. The half-life of tacrolimus is long and variable. Higher rate and extent of absorption of tacrolimus was observed under fasted conditions. Tacrolimus is highly bound (> 98.8%) to plasma proteins, mainly to serum albumin and α-1-acid glycoprotein. Tacrolimus is a low-clearance substance, and the average total body clearance estimated from whole blood concentrations was 2.25 L/h in healthy subjects [3]. In 1999, Bekersky et al. [15] analyzed the whole blood samples of tacrolimus by validated LC-MS/MS method, and the lower limit of quantification was 0.5 ng/mL. The linearity range was 0.5 – 30 ng/mL. In healthy subjects, Prograf 0.5-mg, Prograf 1-mg, and Prograf 5-mg capsules were shown to be linear and bioequivalent when administered as an equivalent dose [3]. 

Generic tacrolimus (Tacpan, Pangraf, etc.) from Panacea Biotec Ltd., New Delhi, India, is approved in the USA, Europe, India, Brazil, and many more countries globally. It is available in multiple strengths, including 0.5, 1, and 5 mg. In some countries, there are further strengths such as 0.25 mg and 2 mg available. This bioequivalence study was undertaken to establish the therapeutic equivalence of the test formulation, developed by Panacea Biotec Ltd. India, containing 1 mg of tacrolimus with the reference formulation of similar strength from Astellas Pharma GmbH, Munich, Germany for registering in the European Union based on EMA guidelines [14]. An equivalent rate and extent of absorption between test and reference formulations would predict an essentially similar effect and safety profile. Panacea Biotec Ltd. developed tacrolimus 1-mg capsules and planned to show that it is bioequivalent to the 1-mg approved formulation on the market (Prograf from Astellas Pharma GmbH). The dose for both test and reference formulations was selected as 5 × 1 mg based on the previous literature, which reported 1 × 5-mg capsule (maximum strength approved on the market) to be bioequivalent to 5 × 1-mg capsules in terms of rate and extent of absorption [15]. The study was further conducted in healthy subjects in order to reduce variability not related to differences between products. Tacrolimus shows greatest rate and extent of absorption under fasting conditions. In addition, EMA also generally recommends conducting all bioequivalence studies under fasting conditions, if feasible, in order to provide the most sensitive test for detection of differences between formulations [16, 17]. Moreover, the label instructions of tacrolimus also suggest that the drug should be administered on an empty stomach or at least 1 hour before or 2 – 3 hours after a meal, to achieve maximal absorption [3]. This study will increase transparency and improve confidence among the transplant community regarding generic formulations of narrow therapeutic index drugs (NTIDs). 

## Materials and methods 

### Subjects 

Subjects were recruited from the clinical facility of Accutest Research Laboratories (I) Pvt. Ltd, Vadodara, Gujarat, India. They underwent screening evaluations to determine eligibility within 21 days prior to dosing of period I. Subjects were included if they were male, aged 18 – 45 years (both inclusive), had body mass index within the range of 18.5 – 30 kg/m^2^, normal physical examination and vital signs, clinically acceptable findings on hemogram, biochemistry tests, serology, urinalysis, 12-lead electrocardiogram (ECG), and chest X-ray (P/A view), agreed not to use any prescription and over-the-counter medications for 14 days prior and during the course of the study, had no history of drug abuse in the past 1 year, were nonsmokers and nonalcoholics, and showed willingness to participate. 

Subjects with known history of hypersensitivity to tacrolimus, any medical or surgical conditions interfering with the functioning of blood-forming organs, gastrointestinal tract etc., history of cardiovascular, renal, hepatic, ophthalmic, pulmonary, neurological, metabolic, hematological, gastrointestinal, endocrine, immunological, or psychiatric diseases and bleeding tendency were excluded from the study. In addition, subjects who had participated in a clinical drug study or bioequivalence study or had donated blood within 90 days prior to the present study or had a history of malignancy or other serious diseases or were found positive for human immunodeficiency virus, hepatitis B, hepatitis C, breath alcohol test before check-in for each study period, drugs abuse done before check-in for each study period, had any contraindication to blood sampling or difficulty in accessibility of veins, or had a history of difficulty in swallowing capsule(s) were excluded. 

Moreover, subjects were also not included if they refused to abstain from a) food for at least 10.00 hours prior to study drug administration until at least 4.00 hours postdose in each study period, b) fluid for at least 1.00 hour prior to study drug administration until at least 1.00 hour postdose in each study period (except 240 ± 2 mL of water given during administration of study drug), c) the use of xanthine-containing food or beverages (chocolates, tea, coffee, or cola drinks) or fruit juice/grapefruit juice and any alcoholic products for 48.00 hours prior to dosing for each study period, and d) consumption of tobacco products 24.00 hours prior to dosing. 

All subjects were confined within the facility for at least 12.00 hours prior to dosing until 24.00 hours postdose in each study period. They visited the study center at 36.00, 48.00, and 72.00 hours postdose during each study period for ambulatory blood sample collection. 

### Study design 

The bioequivalence assessment was performed at the Clinical and Bioanalytical Facility of Accutest Research Laboratories (I) Pvt. Ltd., Vadodara, India, under fasting condition. The trial was designed as a randomized, open-label, balanced, two-treatment, two-period, two sequence, single-dose, truncated, crossover study with a washout period of 19 days between phases. The protocol was approved by the Eupraxia Centre for Clinical Excellence LLP Ethics Committee, Vadodara, Gujarat, India (ECR/149/Indt/GJ/2014). 

This study was conducted as per the principles of the Declaration of Helsinki and adhered to the good clinical practices, EMA regulations, and relevant national laws and regulations. All subjects received information from a qualified medical officer or his/her designee about the study and the risks involved. A written informed consent was obtained from each subject, before initial screening procedures, before their inclusion in the study. The subjects were given a copy of the duly signed informed consent form for their information. Eligible subjects were allotted a subject number to maintain the confidentiality of their identity. 

### Treatment and randomization 

Eligible subjects received a single oral dose of 5 × 1-mg capsule of tacrolimus either test (A; Tacpan from Panacea Biotec Ltd. (Batch Number: 4604508)) or reference (B; Prograf from Astellas Pharma GmbH (Batch Number: 1D5913C)) formulation per randomization schedule (sequences AB or BA) in each study period with water (240 ± 2 mL) at ambient temperature in a sitting position. A mouth-check was done immediately after drug administration to assess the compliance to this procedure. The order of receiving the test or reference drug for each subject during both periods was determined according to the randomization schedule, which was generated using the PROC PLAN program on SAS version 9.2. Randomization was carried out in blocks, ensuring an equal number of subject allocation to sequence, both within the block and overall, which maintained random allocation of drugs over the period and sequence. 

### Blood sampling 

The sampling schedule was planned to provide an adequate estimation at maximum concentration (C_max_) and to cover the blood concentration-time curve long enough to provide a reliable estimate of the extent of absorption. A total of 23 blood samples (3 mL per sample) were collected in each study period. Blood samples were collected in K_2_-EDTA vacutainer at predose (00.00 hours) (collected within 01.00 hours prior to dosing) and at 00.25, 00.50. 00.75, 01.00, 01.25, 01.50, 01.75, 02.00, 02.50, 03.00, 03.50, 04.00, 05.00, 06.00, 08.00, 10.00, 12.00, 16.00, 24.00, 36.00, 48.00, and 72.00 hours postdose within 2 minutes of scheduled sampling time. Subjects remained seated for the first 02.00 hours postdose except for any procedural reason in each study period. After that, the subjects were allowed to engage only in normal activities, avoiding strenuous physical activity. An ambulatory blood sample was to be collected up to 04.00 hours from the scheduled time of blood sample collection. If the subject reported for blood sample collection beyond 04.00 hours, the blood sample was not to be collected, and subject was to be requested to come for the next ambulatory blood sample (if any). The subjects returned to the facility 1 day prior to the subsequent dosing after a washout period of 19 days. Approximately 166 mL (including 20.00 mL for clinical laboratory tests (for prestudy and poststudy) and 8 mL as volume discarded during the use of intravenous cannula) of blood was withdrawn from each subject. 

### Bioanalytical methods 

Analysis of whole blood samples of subjects, for tacrolimus, was done by a validated liquid chromatography-mass spectroscopy (LC-MS/MS) analytical method. The validation procedure was performed in compliance with EMA guidelines [18]. The blood samples were kept in a wet ice bath after collection. Whole blood samples were transferred to appropriate-sized polypropylene screw top (previously labelled with study code and sample code) biological sample storage vials in duplicate (one aliquot as control sample and one aliquot for analysis, the aliquot for analysis contained ~ 1.5 mL of blood) and were placed in a deep freezer maintained at –70 ± 10 °C. Both sets of analytical and control samples were transferred to the sample storage area in a zip lock bag, placed in a thermal box containing dry ice, and were further stored in the deep freezer maintained at –70 ± 10 °C. 

The analytical method validation included 0.300 mL of whole blood samples and extraction by solid phase extraction. Tacrolimus 13CD2 was used as internal standard for tacrolimus. The lower limit of quantification was 1.003 ng/mL. The linearity range of tacrolimus was 1.003 – 99.944 ng/mL, which was enough to quantify the expected concentration range of tacrolimus in subjects’ whole blood samples with the proposed dose. Stability was sufficient to cover all study-related procedures. 

### Statistical analysis 

Considering intrasubject coefficient of variation (CV) of ~ 11.00% for tacrolimus and assuming the “test to reference” null ratio to be varying by 5% for AUC of tacrolimus as being the more difficult parameter to achieve presentation of bioequivalence with narrower acceptance criterion (90.00 – 111.11%), 52 subjects were expected to provide 80% power at 5% level of significance so as to meet bioequivalence criteria. Expecting certain dropouts and/or withdrawals, a sample size of 56 subjects was considered. 

The actual blood sampling time points were considered for the calculation of pharmacokinetic parameters using SAS 9.2. Analysis of variance (ANOVA) was performed on the ln-transformed pharmacokinetic parameters C_max_ and AUC from time 0 to the 72.00 hours (AUC_0–72_) at α-level of 0.05. The ANOVA model included sequence, subjects nested within sequence, period, and treatment as fixed factors. A separate ANOVA model was used to analyze each of the parameters. All effects were tested from the ANOVA model. 

Each ANOVA also included calculation of least-square means, adjusted differences between formulation means, and the standard error associated with these differences. The geometric least square mean ratios of the test and reference formulation and its 90% confidence interval for pharmacokinetic parameters C_max_ and AUC_0–72_ were computed, and bioequivalence was concluded if the confidence interval fell within the acceptable range of 80.00 – 125.00% for C_max_ and 90.00 – 111.11% for AUC_0–72_ for tacrolimus. 

## Results 

### Demographic characteristics 

A total of 56 subjects were enrolled in this study. Of these, 4 subjects dropped out due to personal reasons; 1 at ambulatory sample visit, 48.00 hours postdose of period-I, and 3 in period-II. The remaining 52 subjects completed both study periods and were included in the pharmacokinetic and statistical analyses. The mean (standard deviation (SD)) age, weight, and height of the overall population was 31.31 (7.03) years, 60.31 (7.17) kg, and 166.44 (5.25) cm, respectively (Table 1). 

**Figure 1.**
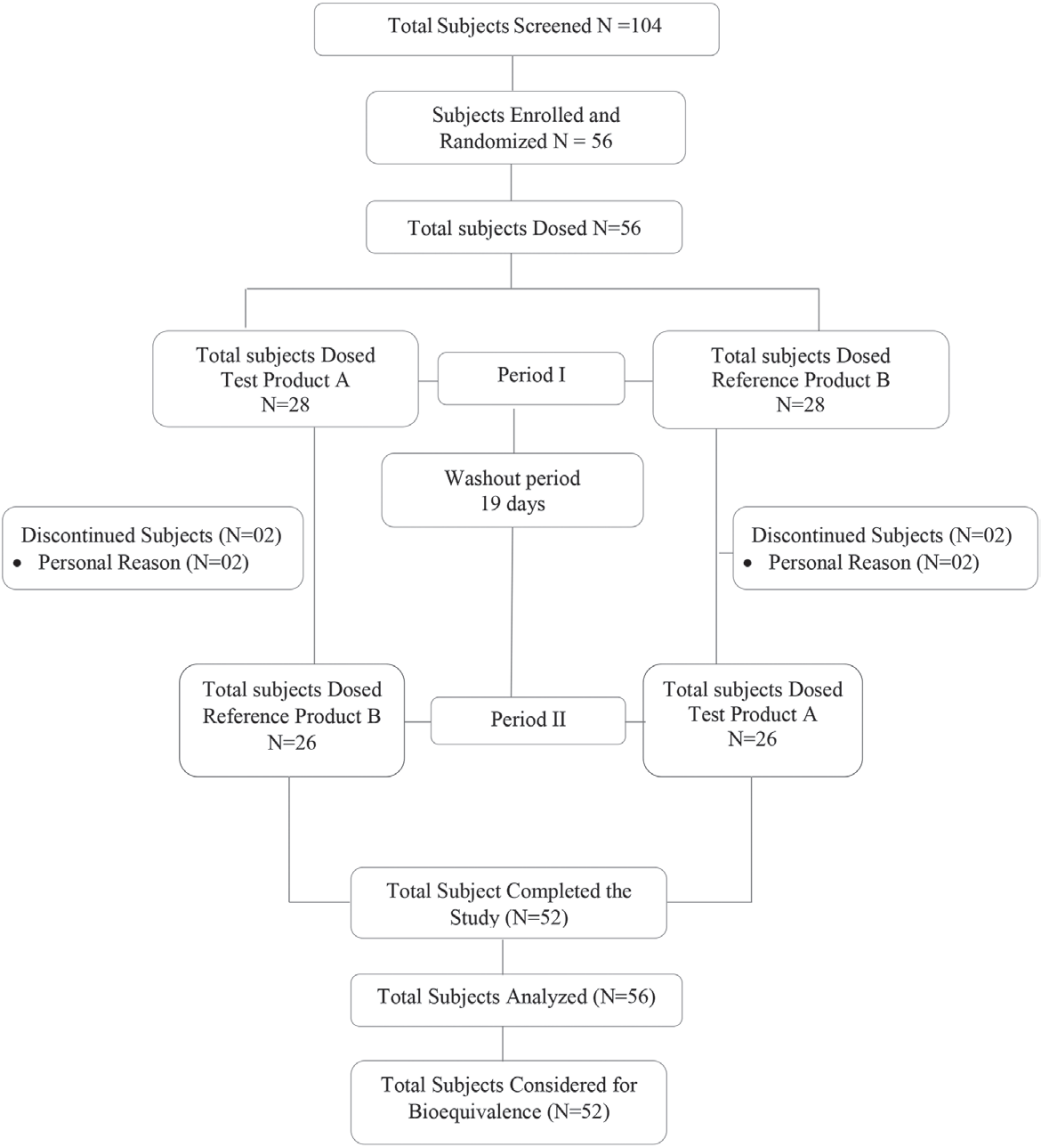
Subject disposition.

A consolidated standards of reporting trials (CONSORT) flow diagram is provided in Figure 1. The duration of the study was 44 days, including screening, washout period of 19 days between each study period, and date of last follow-up. 

### Pharmacokinetic evaluation 

**Figure 2.**
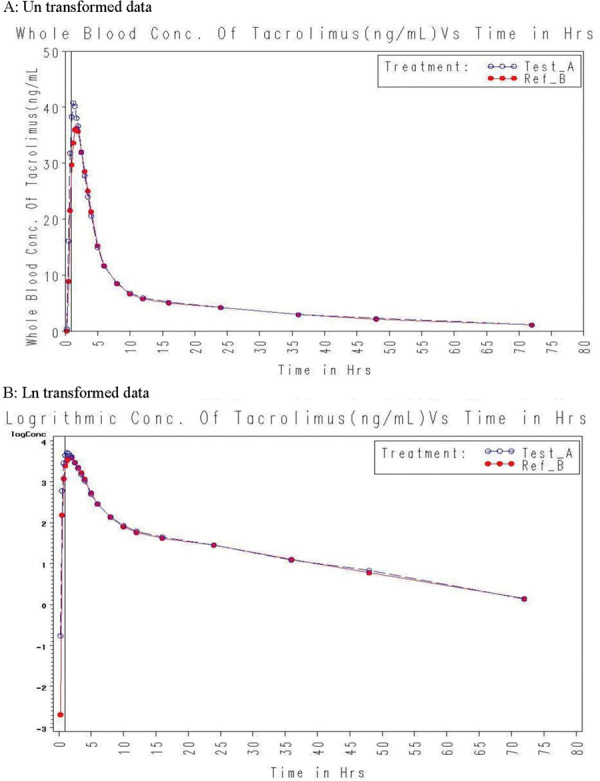
Comparison of tacrolimus time vs. mean concentration curve of test and reference formulations (A: Un transformed data and B: Ln transformed data).

Figure 2 provides the mean whole blood (± SD) concentration of the test and reference formulations of tacrolimus at each pharmacokinetic time point. Four subjects in period 1 and 1 subject in period 2 did not report for ambulatory sample visit and their ambulatory blood samples were missing. Pharmacokinetic and statistical results were reported along with missing samples. The pharmacokinetic parameters of subjects are summarized for both test and reference formulations in Table 2. The arithmetic mean (SD) C_max_ for the reference and test formulations was 40.62 (11.30) and 46.20 (10.73) ng/mL, respectively. For reference and test formulation, the AUC_0–72_ was found to be 348.34 (156.41) and 361.04 (158.71) ng×h/mL, respectively, while the median (range) of t_max_ was 1.38 (0.75 – 4.00) and 1.50 (0.75 – 3.50) hours, respectively. The graphic representations of mean blood concentration-time profiles and mean logarithmic blood concentration-time profiles of the test and reference formulations of tacrolimus are shown in Figure 2. 

### Statistical evaluation 

No subject was detected as an outlier as per Lund test. The geometric least-square mean ratio and 90% CI for pharmacokinetic parameters of C_max_ was 115.07% (95% CI: 109.81, 120.59) and AUC_0–72_ was 103.78 (95% CI: 97.40, 110.58). The intrasubject and intersubject variability was 14.33% and 22.01% for C_max_, and 19.49% and 44.35% for AUC_0–72_, respectively. The power for the C_max_ and AUC_0–72_ was 100.00% and 99.99%, respectively. In an analysis for tacrolimus, no significant sequence, period, and treatment effects were observed for ln-transformed pharmacokinetic parameter C_max_ and AUC_0–72_, except for treatment effect for C_max_. The results indicate that the test formulation was bioequivalent to the reference formulation with respect to C_max_, which was within the bioequivalence range of 80.00 – 125.00% and the AUC_0–72_, which was within the bioequivalence range of 90.00 – 111.11% (within NTID range as per EMA guidelines) (Table 3). 

### Safety evaluation 

Both test and reference formulations were well tolerated. One adverse event of elevated levels of total white blood cells (12.2 × 10^3^/mm^3^) was noted during the poststudy laboratory assessment. This event was mild, possibly related to the study medication but was not resolved since the subject did not appear for adverse event follow-up due to personal reasons. 

No serious or significant adverse event was observed during the entire course of the study. 

## Discussion 

Despite significant market penetration of generic tacrolimus, many clinicians are showing their reluctance to substitute generic tacrolimus over the branded products, particularly due to the narrow therapeutic range and high inter- and intrasubject variability [19]. Due to the very small margin between a safe and a lethal dose, drugs with a narrow margin of safety that may not be satisfied with current general standard bioequivalence range of 80.00 – 125.00% must be dosed carefully [20]. The EMA suggests that the acceptance interval for AUC should be tightened to 90.00 – 111.11% in specific cases of products with a narrow therapeutic index. In cases where C_max_ is of particular importance for safety, efficacy, or drug level monitoring, the 90.00 – 111.11% acceptance interval should also be applied for this parameter [14]. In our study, the 90% confidence interval for the AUC was considered as 90.00 – 111.11%, and for C_max_ 80.00 – 125.00% was used. 

On the other hand, switching to generic immunosuppressants may lead to a significant reduction in out-of-pocket cost and may be an attractive alternative, given that a lifelong therapy is required for long-term graft survival. Generic formulations can provide the necessary flexibility to provide the variable dosing needed in transplantation patients, based on body weight [14]. In view of this, Panacea Biotec Ltd. India has developed a generic formulation of tacrolimus 1-mg capsule and demonstrated the bioequivalence with the reference formulation, Prograf from Astellas Pharma GmbH, Germany, to register it in the European Union. To meet the requirements of bioequivalence, the study was conducted in healthy subjects in accordance with EMA guidance. Consistent with administration guidance, as given in the summary of product characteristics, bioequivalence was examined following administration of tacrolimus in the fasting condition as the rate and extent of tacrolimus absorption is higher under fasted conditions. As suggested by EMA guidelines on the investigation of bioequivalence, the reliable estimate of tacrolimus from its immediate-release formulation can be obtained with the truncated study design (i.e., 72 hours) since tacrolimus has a longer T_1/2_ and requires a longer sampling period, therefore, it is not considered necessary for full sampling [14]. In 2011, EI-Tahtawy et al. [21] investigated the use of truncated AUC (like AUC_0–72h_) as a surrogate for AUC_0–∞_ for drugs with long half-lives (≥ 30 hours) and did not find opposing results. Hence, we chose a truncated study design (0 – 72 hour sampling) for the study [21]. 

The results of the current single-dose, randomized, open-label, two-way cross-over truncated study indicated that the 1-mg test formulation of tacrolimus (when administered at a dose of 5 mg) met the regulatory definition of bioequivalence to the reference formulation in accordance with EMA guidelines in this selected population. The bioequivalence shown in our study implies that similar tacrolimus exposure is achieved when individuals are switched between these tacrolimus products, in patients with organ transplant. 

Our results support many other pharmacokinetic studies where generic tacrolimus formulations have been reported effective and safe in immunosuppression in transplant patients [22, 23, 24, 25]. Alloway et al. [22] showed bioequivalence between innovator and two generic tacrolimus, as well as between two generic products, following FDA bioequivalence metrics, in individuals with a kidney and liver transplant. Another study comprising 68 kidney transplant recipients showed a similar pharmacokinetic profile (AUC C_0_, C_max_, or t_max_) for generic and branded tacrolimus as per both USFDA and EMA guidelines [24]. Further to this, a retrospective matched group analysis between generic tacrolimus and Prograf in renal transplant patients reported that generic tacrolimus was not different from the branded drug (Prograf) even after 1-year post-transplantation. Both generic formulation and branded drug showed good efficacy with comparable safety, and the drug concentrations did not differ [26]. Marfo et al. [19] (2013) also performed a study on renal transplant patients who were receiving prograf brand-name tacrolimus and were switched to generic tacrolimus. The results reported that the generic tacrolimus was safe and effective and did not confer negative clinical outcomes [19]. 

In a prospective study, clinically stable patients after liver transplantation were converted from the innovator formulations of tacrolimus (Prograf) and mycophenolate mofetil (Cellcept) to the generic formulations of tacrolimus (Tacpan) and mycophenolate mofetil (Mowel). Patients were followed-up for 6 months. Results were compared retrospectively to age- and sex-matched controls treated with the original brands, whereby both generic formulations and branded drugs showed good efficacy with comparable safety. In the matched-pair analysis of tacrolimus trough level/dose ratio, no significant difference was found between Tacpan/Mowel and Prograf/Cellcept groups. Intraindividual analysis of costs revealed a considerable cost reduction in the Tacpan/Mowel group. In summary, the use of the bioequivalent generics tacpan/mowel in stable liver transplantation patients, while as safe and as effective, is significantly more cost-effective in comparison to the original drugs [27]. 

Bekersky et al. [15] (1999) examined the bioequivalence of 1 mg Prograf (dosed as 1 mg × 5 capsules) vs. 5 mg Prograf (dosed as 5 mg ×1 capsule). The 90% confidence intervals were 90.5 – 101.9% for C_max_ and 87.1 – 101.7% for AUC_0–t_. The authors concluded that since all these values fall within the range of 80 – 125%, the 1-mg and 5-mg tacrolimus capsules were bioequivalent with respect to both rate and extent of absorption. In 1998, Bekersky et al. [28] showed 0.5-mg Prograf capsules (dosed as 0.5 mg × 6 capsules) to be bioequivalent to the 1-mg Prograf capsules (dosed as 1 mg × 3 capsules) as the 90% CI for 0.5-mg and 1-mg capsules were 92.6 – 115% for C_max_ and 93.1 – 113% for AUC_0–t_ [15, 28]. Results from these studies suggested that the variability in tacrolimus pharmacokinetics observed in early studies most likely was attributable to the analytic method used at that time rather than an intrasubject/intraformulation variability in absorption. 

The intrasubject CV observed in our study was below 30% and, therefore, a standard two-way crossover design should allow proper comparison between the test and reference formulations. As an NTID, TDM for tacrolimus is recommended in the routine clinical setting to prevent blood levels from crossing the upper limit for toxicity or the lower limit for effective immunosuppression in patients with organ transplantation [29]. The consequences of over- and under-dosing are of major clinical importance and can substantially affect clinical outcome. In clinical practice, the therapeutic range (whole blood trough levels) of tacrolimus in liver transplant recipients should be generally in the range of 5 – 20 ng/mL in the early post-transplant period. The target range of whole blood trough levels in kidney and heart transplant patients should be 10 – 20 ng/mL. However, the blood concentrations should generally be in the range of 5 – 15 ng/mL in liver, kidney and heart transplant recipients during maintenance therapy [3]. The European Consensus Conference on Tacrolimus TDM proposed AUC targets for tacrolimus between 150 and 200 ng×h/mL [30]. Further studies analyzing the AUC-based TDM of tacrolimus are required. 

Despite reluctance to the use of generic substitution, studies have shown that the use of generic version of NTIDs is as effective and safe as their brand name versions [31]. A recent survey on pharmacists’ substitution beliefs and practices on generic versions of NTIDs revealed that 87% and 94% of the pharmacists perceived generic NTI drugs to be as effective and safe as their brand-name versions, respectively [32]. To be on the safe side, clinicians should be aware of possible changes in the efficacy while switching from a brand name to other generics, particularly when careful titration and patient monitoring is required. The therapeutic interchange in patients with organ transplant who are stabilized on a drug therapy regimen should be closely monitored for drug levels and/or ECG or other pharmacodynamic markers to assure the desired clinical response [31]. Moreover, caution is warranted since narrowing the bioequivalence boundaries could not address the high interindividual variability or differing pharmacokinetic properties in different patient populations. 

The study has some limitations. Firstly, the current study determined the bioequivalence of the test formulation as per the requirement of the EMA for abbreviated new drug application submission, in young healthy subjects, which certainly does not equate to the patient population. Secondly, transplanted patients generally have comorbidities and are treated with several drugs and are quite different from the population of general bioequivalence studies, which are generally performed in healthy subjects. Thirdly, only specific pharmacokinetic parameters, including C_max_, t_max_, and AUC_0–72_ hours were calculated in the study. We did not evaluate other pharmacokinetic parameters like T_1/2_, t_lag_, t_last_, AUC_expol_ since this was not a regulatory requirement. 

The behavior (like therapeutic efficacy, safety, and clearance) of the active ingredient is completely independent of the drug formulation after absorption across the gut mucosa, and the formulation is the only product-specific component that can be different between the innovator and the generic product. Hence, it is more difficult to identify possible differences between two formulations in a patient population with significant variability than in rigorously controlled studies in healthy individuals [12]. Further studies are needed to evaluate the efficacy and safety of generic drugs in the early and late phase after transplantation. 

## Conclusion 

In conclusion, the test formulation (tacrolimus 1-mg capsules) of Panacea Biotec Ltd. India met the European regulatory definition of bioequivalence as compared with Prograf (tacrolimus 1-mg capsules) of Astellas Pharma GmbH, Germany after a single-dose administration under fasting conditions and was well tolerated. The switch to the test formulation seems to be safe and effective and may reduce out-of-pocket cost and economic burden. 

## Acknowledgment 

We thank GCE Solutions for assisting in preparation of the manuscript. 

## Funding 

The study was fully funded by Panacea Biotec Limited. 

## Conflict of interest 

All authors are employees of Panacea Biotec. The principal investigator of the study had no financial interests in the product or the manufacturer but received research funding to undertake the study. 

**Table 1.** Baseline features of subjects.

Variables	N = 56 (all enrolled subjects)	N = 52 (considered for bioequivalence)
	Mean ± SD (Max : Min)	
Age (year)	31.43 ± 6.86 (20 : 45)	31.31 ± 7.03 (20 : 45)
Height (cm)	166.29 ± 5.31 (153 : 180)	166.44 ± 5.25 (153 : 180)
Weight (kg)	60.23 ± 6.97 (50 : 80)	60.31 ± 7.17 (50 : 80)
BMI (kg/m^2^)	21.79 ± 2.41 (18.56 : 29.38)	21.77 ± 2.48 (18.56 : 29.38)

BMI = body mass index; SD = standard deviation; N = number of subjects.

**Table 2.** Pharmacokinetic parameters of tacrolimus estimated for both the test and reference formulations.

Formulations		PK parameters		
		C_max _(ng/mL)	AUC_0–72 _(ng×h/mL)	t_max_*(hours)
Test formulation A (N = 52)	Arithmetic Mean ± SD (CV (%))	46.20 ± 10.73 (23.22)	361.04 ± 158.71 (43.96)	1.38 (0.75 – 4.00)
Reference formulation B (N = 52)	Arithmetic Mean ± SD (CV (%))	40.62 ± 11.30 (27.81)	348.34 ± 156.41 (44.90)	1.50 (0.75 – 3.50)

*For t_max_ median and range are reported. CV = coefficient of variation; PK = pharmacokinetic; SD = standard deviation. Test formulation A: generic tacrolimus; reference formulation B: innovator tacrolimus.

**Table 3.** Geometric mean and 90% confidence interval based on the least squares mean for test and reference formulation (N = 52).

Parameters	Geometric mean*		% Ratio	90% Confidence interval	
	Test (A)	Reference (B)	A/B	Lower limit	Upper limit
AUC_0–72_	327.01	315.09	103.7831	97.4019	110.5824
C_max_	44.99	39.10	115.0745	109.8079	120.5938

*obtained by ANOVA for the ln-transformed pharmacokinetic parameters C_max_ and AUC_0–72_. Test formulation A: generic tacrolimus; reference formulation B: innovator tacrolimus.
